# Engineering localized injury: a 3D microfluidic platform approach for ex vivo tissue interrogation

**DOI:** 10.1007/s00216-025-06245-9

**Published:** 2025-12-06

**Authors:** Colby E. Witt, Lauren M. Delong, Maria K. Kristinsdottir, Alexandra K. Brooke, Ashley E. Ross

**Affiliations:** Department of Chemistry, University of Cincinnati, 312 College Dr., 404 Crosley Tower, Cincinnati, OH 45221-0172 USA

**Keywords:** Ischemia, Neurochemicals, Tissue stimulation, Microfluidics, Microfabrication

## Abstract

Over the years, interrogating and replicating biological events ex vivo and in vitro has proven to provide a breadth of knowledge into various mechanisms of diseases; however, there remains a challenge in understanding the onset of disease within tonic, localized environments over time. Microfluidics provides an alternative approach that can be coupled with many methods to better replicate specific localized and prolonged biological events. In this work, we introduce a novel 3D-printed device that significantly improves upon previous microfluidic approaches aimed at maintaining high spatial resolution and sustainment of delivery, ultimately enabling us to examine localized injuries within an ex vivo model with higher precision and control. This work focuses on focal ischemia; however, it should be noted that the device is applicable to any event that needs high spatial resolution and sustained fluidic delivery. We demonstrate that tissue remains viable on this platform and that we are capable of achieving exquisite fluidic control, not affordable from standard lithographic techniques. Additionally, we incorporate real-time neurochemical monitoring to demonstrate rapid and robust changes in dopamine signaling at the site of focal ischemia, further validating our platform for localized injury studies. This device fills a critical gap in the existing literature by significantly improving upon the capabilities of localized tissue stimulation and further enhances our understanding of local physiological changes during ischemic events, providing a critical advance in biomedical tools for measuring at localized regions within tissue.

**Graphical Abstract:**

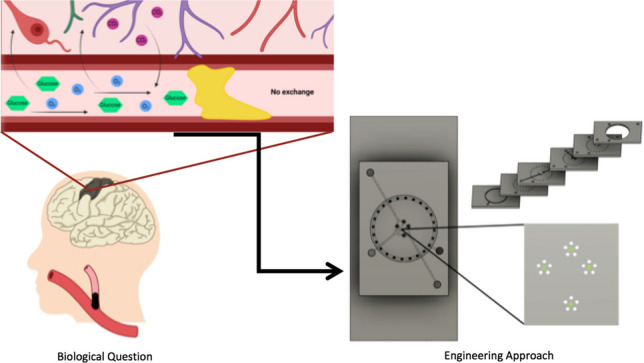

**Supplementary Information:**

The online version contains supplementary material available at 10.1007/s00216-025-06245-9.

## Introduction

Biological processes often occur at highly specified and localized sites within the body, where the intricate interactions of cells, tissues, and molecules are tightly regulated to maintain homeostasis and respond to external stimuli [1, 2]. This spatial specificity is essential for understanding complex physiological and pathological processes. However, replicating or probing these localized events for research and therapeutic purposes presents significant challenges.

Traditional approaches to studying biology, such as system-wide drug delivery or broad-spectrum pharmacological interventions, often fail to capture the complexity and precision of localized biological activities, leading to less targeted outcomes and systemic side effects [3, 4]. Moreover, many methods for probing tissue-specific mechanisms are either too invasive or lack the ability to reach the right site at the right time with the necessary precision. In this context, the development of innovative technologies that enable the local, targeted delivery of therapeutics or experimental agents represents a significant breakthrough [5].

Over the years, ex vivo models have been a key step in understanding the neurochemical underpinnings of disease states [6–8]. Though there are many advantages to studying slice models, in vivo measurements keep brain circuits intact and often differ from data obtained from ex vivo brain slices [9]. The conditions in ex vivo studies, fully controlled by researchers, differ markedly from the dynamic in vivo environment, necessitating cautious interpretation of findings [9]. In vivo studies incorporate the biologically relevant cell signaling dynamics; however, when trying to isolate these localized and dynamic events, it remains challenging within an in vivo environment. Therefore, there is a need for a middle-out approach which maintains the complexity of organ-level multi-cell signaling interactions yet renders the organ experimentally accessible for localized probing and interrogation.

Microfluidics presents a unique opportunity to explore this necessary middle-out approach due to its capability to be coupled with biological specimens. Platforms are often engineered to control tissue agitation or assault (1–1000 µm scale) with high spatial resolution [10–12]; microfluidics has also been used extensively to stimulate tissue locally [5, 13–17]. Great strides have been achieved in mimicking the localized sight of ischemia with microfluidic platforms; however, the duration of spatial containment is currently limited to roughly one minute [14]. It is imperative to extend our capabilities for longer-term localized probing due to a host of biologically relevant events that occur on much longer time scales.

Our lab has made significant contributions to this area over the last several years, designing microengineered platforms for localized delivery to tissue [14, 18]. Despite these advancements, our ability to maintain high spatial resolution over times greater than a few minutes was limited due to the inherent design of the device. With soft lithography, the design capabilities are somewhat limited due to the inability to adequately capture complex three-dimensional structures on-chip. To circumvent this inherent limitation to lithographic approaches, we have adopted a 3D-printed approach. The use of 3D printing significantly widens our design capabilities, ultimately lending to significantly improved experimental control within tissue environments. This new device enables sustained, spatially resolved delivery of injury in ex vivo tissue slices and shows a significant improvement over previously published work focused on localized tissue stimulation [14]. Overall, this device bridges a critical gap in the literature and will directly impact our ability to understand local physiology during injury when coupled with fast-voltammetric techniques. Our application of choice within this work is focused on focal ischemia; however, it must be stressed that this device design and approach are applicable to many biological processes beyond brain ischemia.

Ischemic strokes, characterized by the abrupt cessation of blood flow to parts of the brain, account for a staggering 87% of all stroke cases [19–21]. These events impair quality of life by affecting speech, fine motor skills, and causing brain damage [22–24]. Despite the development of new therapies, our understanding of brain recovery mechanisms post-ischemic stroke is limited [25–27]. Investigation of brain changes in real time can help identify biomarkers of interest and improve therapeutic targets [28, 29]. The hippocampus’s CA1 region is particularly susceptible to ischemic stroke damage due to its dense glutamatergic and dopaminergic innervation [30–33]. During ischemic events, the release of these neurotransmitters leads to excitotoxicity and oxidative stress, causing localized cell death, ultimately triggering spreading depolarization, resulting in widespread brain damage [34, 35]. Using our fabricated 3D platform, this device features strategically placed channels with entry ports to administer a liquid stimulus precisely to tissue ex vivo, ensuring targeted and sustained delivery for significantly enhanced experimental capability. Prior reports have described devices to achieve similar goals; however, we present here a brand new containment strategy [5], which we call the “floret” design. This unique design and device structure can be easily achieved by 3D printing and is not compatible with standard soft lithography approaches, which makes it a directly translatable design for multiple laboratories. In this configuration, a circular array of ports directs the buffer flow, enabling controlled stimulation by adjusting the flow rate. The device incorporates an open culture well, accommodating tissue slices for extensive analysis. In this particular work, brain slices are coupled on-chip with fast-scan cyclic voltammetry (FSCV) recording to measure subsecond fluctuations of neurochemical signaling at localized sites of focal ischemic injury. The key innovation of this platform lies in its ability to achieve sustained spatial containment of a delivered stimulus for over an hour, significantly extending the duration compared to previously reported microfluidic systems. Additionally, this device design can be easily applied to probe a variety of biomedical questions, specifically those that seek to understand local physiology in intact tissue sections. Not only this, but this unique design allows probing various regions of tissue simultaneously due to the strategically placed delivery ports of the stimulus and supports dual-drug studies.

## Experimental methods

### Device fabrication and assembly

The device printer file was designed using Autodesk Fusion 360 (2021) software. The device was fabricated via a CADworks H-50 3D printer (CADworks3D, Ontario, CA) with the clear, biocompatible resin (CADworks3D, Ontario, CA). The device was printed in 30 µm sections. The printed devices were removed from the 3D printer stage and cleaned in a warm, soapy water bath. After soaking (10 min), soapy water was also perfused through all channels to ensure no excess resin was left in the channels. The device was then cured using a UV light box for 10 s on the top and bottom of the device; the device was rinsed once more before experiments were performed.

**Fig. 1:**
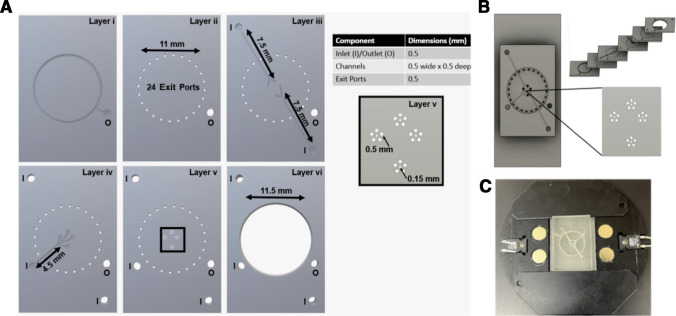
Microfluidic 3D-printed platform designed for localized and sustained chemical delivery to tissue ex vivo. **A** All layers of the chip exploded to depict the delivery channels and exit port placements. Ports denoted “O” are outlets where fluid is pulled, and ports denoted “I” are for tubing inlets for fluid delivery. Layer i (bottom layer) was designed to remove excess fluid from the device to prevent overfilling of the tissue chamber. Layer ii provides ports where fluid is removed. Layer iii contains the channels for delivering stimulus to the stimulus ports. Layer iv houses the channel for normoxia or “normal” buffer to the brain tissue and around the stimulus delivery regions for containment. Layer v comprises the exit ports. “Floret” designed ports were created for optimal stimulus resolution; buffer is distributed to the outside portions of the ring while the stimulus is delivered in the center. A magnified region of the floret design is shown to the right of Panel A. Layer vi (top layer) contains a 2-mm tissue culture well that is open for easy interrogation of tissue onboard the device. **B** 3D rendered model of the assembled device (top view). **C** Image of the 3D-printed device incorporated into a commercial perfusion chamber base for tissue analysis

The bottom layer printed was designed to fit in a standard commercially available perfusion chamber basin (2 mm tall, 24 mm wide, 50 mm long) for experimental practices (perfusion chamber from Warner Instruments). The 3D-printed microfluidic platform is designed into six layers (Fig. 1) with all six layers printed at 0.8 mm tall. Two channel types exist within the device: “normoxia” channels and “ischemia” (or stimulus) channels (Fig. 1 (layer iii)). The stimulus channels are 0.5 mm wide by 0.5 mm deep and are surrounded by “floret” ports (0.15 mm) (layer v) to enable spatial containment of the stimulus of choice. The tissue is kept viable via the buffer delivery layer (layer iv), where we continuously perfuse artificial cerebral spinal fluid (aCSF) to keep the brain viable and in a homeostatic state. Twenty-four exit holes (0.5 mm) and channels (0.5 mm by 0.5 mm) are located at the bottom two layers (layer i and ii) to remove waste and to facilitate the removal of excess fluid from the culture well to eliminate spillage. The top layer is an 11.5 mm circle (diameter) and 2 mm deep that is open to the top for the tissue to reside and be easily accessible.

### Reagents

Unless otherwise noted, all reagents were purchased from Fisher Scientific (Pittsburgh, PA). Brain slice experiments were conducted with artificial cerebrospinal fluid (aCSF). Two types of aCSF were used: “normoxia” and “ischemia” aCSF. The normoxia aCSF was made with 2.5 mM KCl, 1.2 mM NaH_2_PO_4_, 2.4 mM CaCl_2_, 1.2 mM MgCl_2_, 126 mM NaCl, 11 mM d-glucose, 25 mM sodium bicarbonate, and 15 mM tris(hydroxymethyl)aminomethane. The ischemia aCSF was the same recipe except with d-glucose removed. Normoxia aCSF was oxygenated with a 95% O_2_ and 5% CO_2_ mix. The ischemia aCSF was deoxygenated using ultrapure N_2_ gas. Information on Calcein-AM and 2,3,5-triphenyltetrazolium chloride (TTC) chemical preparation is listed in later sections.

### Carbon fiber microelectrode fabrication

The microelectrodes were fabricated using 7-µm T-650 carbon fibers (Mitsubishi Chemical Carbon Fiber and Composites, Sacramento, CA). The fibers were aspirated into a 1.2 × 0.68 mm glass capillary (A&M Systems, Sequim, WA). The capillaries with the fiber inside were pulled using a vertical PE-22 Electrode Puller (Narishige, Tokyo, Japan) to form two carbon fiber microelectrodes. The excess fiber length was cut to 100–150 µm from the glass seal via a scalpel under a microscope. The electrodes were backfilled with KCl for electrical connection. It should be noted that fibers were cut on the day of the experiment. All measurements were made against an Ag/AgCl reference electrode.

### Fast-scan cyclic voltammetry (FSCV)

FSCV at carbon-fiber microelectrodes was performed using a Dagan Chem-Clamp potentiostat (Dagan Corp., Minneapolis, MN) coupled to a UNC breakout box (UNC Electronics Shop, Chapel Hill, NC). High-definition cyclic voltammetry (HDCV) software (UNC at Chapel Hill) with a multifunction I/O device (PC1e-6363, National Instruments, Austin, TX) was used for data acquisition and analysis. The standard “dopamine waveform” was used in all FSCV experiments (−0.4 to 1.3 at 400 V/s, 10 Hz) [36]. A 3 kHz low-pass filter was used, and all data were background subtracted to eliminate nonfaradaic current. Dopamine current was converted to concentration using the slope of a calibration curve, as previously reported [14]. In this experimental paradigm, tissue was placed at the center of the device and anchored by a washer to ensure that the slice would not move during voltametric interrogation. Once on the device, oxygenated aCSF was perfused throughout the device, allowing equilibration of the slice on the platform (20 min). After the equilibration period, baseline dopamine transient events were monitored for 15 min. After the baseline/control data was taken, the fluid was switched within the analyte delivery channel layer (using the Y-channel inlet design), and ischemic buffer was perfused through the local stimulation ports while the rest of the slice was provided with oxygenated aCSF. Over the 30-min period, dopamine transient events were monitored; the first 15 min are reported as “sham” while the last 15 min are reported as “ischemia.

### Brain slice preparation

All animal procedures were approved by the Institutional Animal Care and Use Committee (IACUC) at the University of Cincinnati and were performed in accordance with *The Guide for the Care and Use of Laboratory Animals* (“*The Guide*”) by the National Research Council. Sprague–Dawley rats (male) weighing 170–180 g (Charles River Laboratories, Wilmington, MA) were housed in a vivarium accredited by the Association for Assessment and Accreditation of Laboratory Animal Care (AAALAC) and provided food and water ad libitum. Rats were anesthetized with isoflurane (Henry Shrein, Melville, NY) and euthanized via decapitation prior to the experiment. The brain was immediately removed and placed in ice-cold oxygenated (95% O_2_ and 5% CO_2_) aCSF for rest (~ 2 min). The brain was then dissected to remove the cerebellum and sliced down the midline for mounting onto a Leica stage mount. Sagittal slices of the hippocampus (400 µm thick) were obtained using a Leica VT1000S vibratome (Chicago, IL) set to a speed of 90 and a frequency of 3. The tissue was left for recovery for 1 h in oxygenated, room-temperature aCSF prior to experimentation.

### Validating local and sustained stimulation

A 0.1 mg/mL solution of fluorescein (1 × PBS) (Sigma-Aldrich) was prepared and used to validate localized and sustainable stimulus delivery on-chip. Fluorescein was delivered through the stimulus delivery channels, while PBS was delivered to the channels/floret ports surrounding the stimulus (Fig. 1B). Waste was removed from the device via the waste channels (labeled, Fig. 1). Characterization studies were done in 300 micron-thick 6% agarose slice pads due to their optical transparency and similarity in permeability to tissues [31, 33]. Agarose slices were anchored to the chamber via an 11-mm diameter stainless steel washer (Fastenal). Fluorescent images of stimulus containment within the slice were taken using a Zeiss AxioZoom macroscope (Carl Zeiss Microscopy, Germany) with an Axiocam 506 mono-camera via the GFP (Zeiss filter set # 38) filter cube. Images were taken every 10 s for 1 min, every minute for 9 min, then every 10 min for 50 min. Fluorescein was delivered at a 1 µL/min flow rate, while the buffer was delivered at 10 µL/min (a previously optimized flow rate [14]) using a peristaltic pump (Ismatec Regalo ICC, Cole-Palmer, USA). The intensity of the fluorescence vs. the distance of the line was plotted and analyzed using Fiji (ImageJ). The data were fit with a Gaussian Distribution, similar to prior work [12, 14, 18, 37], and this fit was used to quantitate relative spread over time. From the peak intensity, the half-maximum intensity was quantitated. The distance between the two half-maximum values is defined as the stimulus spread. See electronic supplemental material for example raw data (Figure S1). Note that the microfluidic system was filled with fluid for at least 5 min prior to the experiment to ensure all channels were loaded and care was taken to avoid introducing air during loading.

### Calcein staining

Brain slice viability was assessed using 1 mM Calcein-AM (ThermoFischer, USA) and compared both on and off-chip. The slices were then placed into the wells with the solution and incubated in the dark (room temp) for 20 min. After the incubation period, the slices were rinsed with PBS buffer and imaged with a Zeiss AxioZoom macroscope (Carl Zeiss Microscopy, Oberkochen, Germany) using the GFP filter cube in a small petri dish (AE Bios, Ohio, USA) filled with PBS buffer.

### TTC staining

1X PBS (made from 10X stock) was mixed in a vial with TTC powder (Sigma-Aldrich, St. Louis, MO) at a 2% w/v ratio. The vial was covered in aluminum foil and placed in an incubator for 30 min at 37 °C. All the experimental brain slices were placed on the platform for 30 min while the delivery of the treatment group (normoxia and ischemia, *n* = 4 for each model) was funneled through the delivery ports. After 30 min on the device, the slices were gently brushed and fully submerged in the petri dishes containing the warmed TTC solution. The dish was covered and kept at 37 °C in the incubator (30 min, dark). The slices were taken out from the incubator and washed with the 1 × PBS solution before fixation with 10% formalin neutral buffered solution (Sigma-Aldrich, St. Louis, MO). The slices were left for 24 h in formalin solution. The slices were then washed with 1 × PBS three times and placed on a gelatin-coated glass (75 × 25 mm) slide. The excess liquid around the slices was dabbed using a Kim wipe. The slices were imaged with an AxioZoom macroscope (Carl Zeiss Microscopy, Oberkochen, Germany) with an Axiocam 506 mono-camera. Fourteen-bit images were captured and analyzed in Zen software (Carl Zeiss Microscopy), and analysis for infarct diameter was done using ImageJ software.

### Statistics and figures

All statistical tests were performed using GraphPad Prism 9 (GraphPad Software, Inc., La Jolla, CA, USA). Significance was determined via a confidence level of 95% threshold (*p* < 0.05). Unless otherwise noted, the “*n*” number value represents the brain slice number. The figures were crafted utilizing Inkscape, and original images were created with a BioRender (BioRender.com) subscription.

## Results and discussion

### Device design

Here, we developed a 3D-printed platform that incorporates an open tissue culture well to enable easy access to brain slices with simultaneous local fluidic stimulation from underneath. The device is compatible with real-time neurochemical recording using external electrodes and imaging. Unlike prior work, this platform enables full encapsulation of the stimulus delivery zone through the use of a “floret design” (Fig. 1). The encapsulation of the stimulus port provides significantly improved sustainment of local delivery over time, significantly widening the experimental window used for local tissue probing. For this work, we chose to use this platform to locally administer glucose and oxygen-deprived media to sub-millimeter regions of tissue over time to demonstrate the temporal control over the spatial resolution of delivery. The device is composed of six distinct layers, each engineered to perform a specific function for controlled, spatially resolved fluid delivery to ex vivo tissue. Layer i, the bottom layer, is designed to collect and remove excess fluid from the system, preventing overfilling of the tissue chamber and maintaining stable experimental conditions. Layer ii contains fluid removal ports that interface with Layer i to facilitate active withdrawal of fluid during operation. Layer iii houses the channels responsible for directing the stimulus to the central delivery ports. Layer iv contains a separate channel network that delivers a continuous flow of normoxic buffer to the tissue; this buffer surrounds the stimulus delivery region and plays a dual role: maintaining physiological baseline conditions and has been designed to contain the spread of the applied stimulus. Layer v incorporates exit ports that support continuous fluid flow-through and waste removal. Finally, layer vi, the top layer, features a 2-mm open tissue well that enables easy placement and direct access to tissue slices, making the device eligible to be coupled with other techniques (i.e., imaging). Central to the design is the “floret” configuration, in which peripheral ports direct buffer flow in a circular pattern around a central stimulus port. This configuration enables precise spatial control of stimulus delivery by confining the stimulus to the center while the surrounding buffer flow prevents lateral diffusion, achieving fine resolution and reproducibility in localized tissue stimulation.

**Fig. 2:**
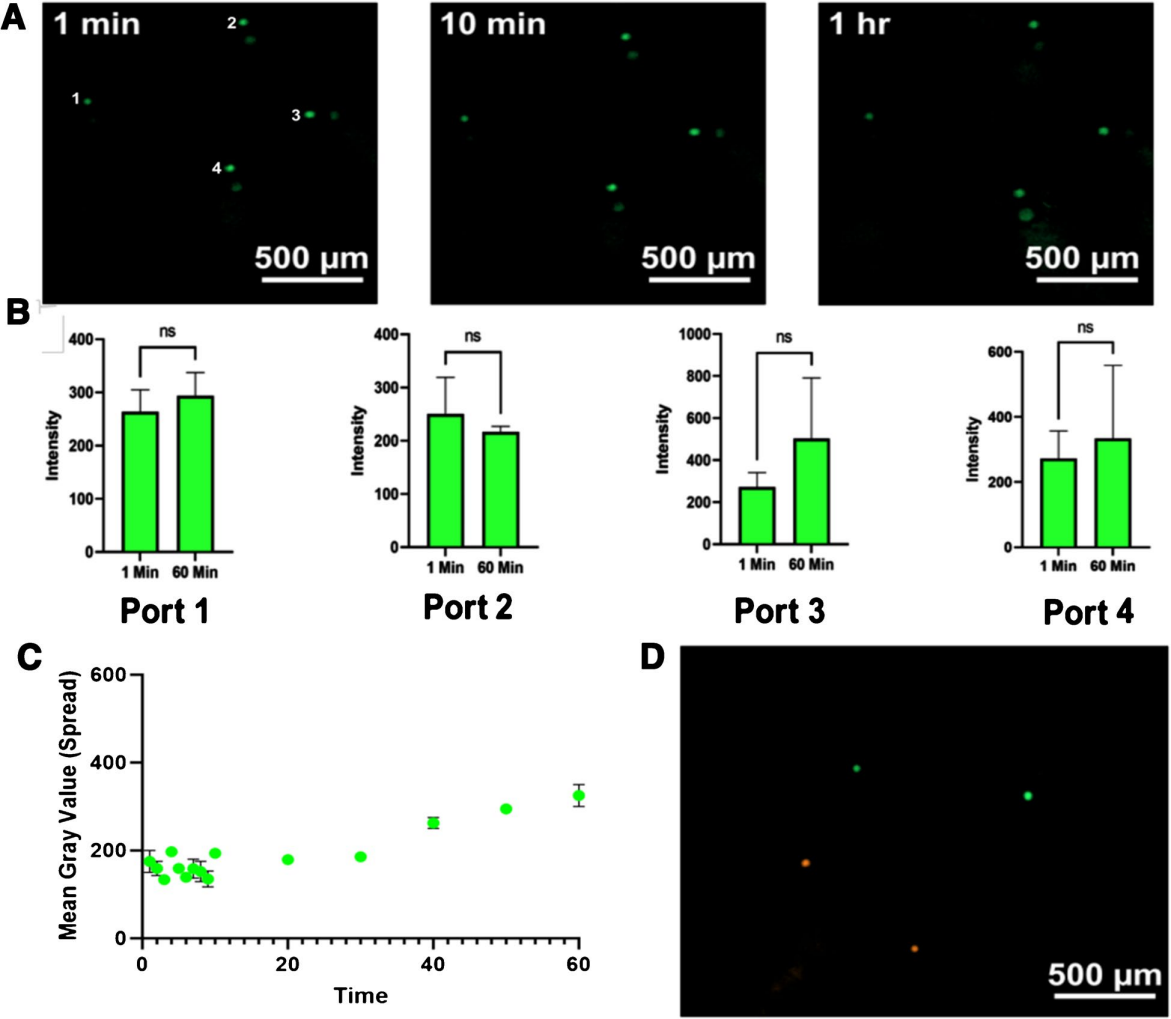
Fluorescein delivery to agarose slices is focused and maintained on-chip. **A** Images at 1-, 10-, and 60-min marks show, at a 1:10 flow ratio, a pinpoint spatially contained fluorescent stimulus. Port numbers are labeled in the 1-min image. **B** Relative spread intensity at the 1- and 60-min points at all four ports. The average spread at the 1-min mark is 264.1 ± 27 µm while the average spread at the 60-min mark is 304 ± 45. **C** Average across four ports showing that there is relative stability in the containment of the stimulus over time. **D** Dual delivery of fluorescein and rhodamine on-chip to two stimulus ports each. This experimental design demonstrates the ability to target multiple regions simultaneously and with high spatial control. The current orientation was chosen to better illustrate the connection between the ports and the common buffer lanes (*n* = 5 for all experiments)

The dimensions of the device were carefully engineered to match our specific experimental needs (localized ischemia delivery to the brain); however, it is important to note the customizability with ease that 3D printing affords. The design concept can be manipulated easily to fit any localized tissue delivery needs. Here, our device contains channels 0.5 mm wide by 0.5 mm deep, and all exit ports are 0.5 mm in diameter, while the inlets/outlets to the buffer delivery channels are 1.57 mm in diameter to accommodate the tubing attached (Fig. 1). The center stimulus port is smaller, only 0.1 mm in diameter, to better mimic the size of an artery in a rat brain where an ischemic occlusion would occur [20]. Again, these design features can easily be manipulated to fit the specific intended application. The culture well in layer vi is 11.5 mm in diameter by 2 mm deep to accommodate the diameter of the brain slices on the device. The stimulus delivery layer (layer iii) has strategically placed ports to deliver an ischemic stimulus to multiple sites across the brain tissue. Here, our design was constructed to facilitate delivery to multiple sub-regions within the hippocampus region: notably, the CA1, CA3, and dentate gyrus (tissue is fixed to the platform via a metal disk previously described). These ports could be easily redrawn in different regions to suit the experimental goals. The buffer delivery layer (Layer iv) was designed to enable oxygenated media to the slice to keep the tissue healthy on-chip. The spatial containment layer (layer v) was designed to deliver the analyte in the center of a “floret” to facilitate finely resolved stimulation from layer iii; all solutions are circulated by the combination of these layers. The inlets were labeled “I” on the device design layout (Fig. 2A). All waste is pulled via layers i and ii to outlet (O) found on the edge of the device (Fig. 2A).

The flow rate of fluid delivery is easily controllable using external perfusion pumps. Previously, our lab optimized a flow rate ratio of 1:10 (stimulus:buffer) for optimal spatial containment and limited shear stress in brain tissue [14]. Here, we adapted this flow ratio for the remainder of the device characterization; however, because flow rates are easily tunable, this parameter can be easily manipulated for the intended application. Waste was pulled from the middle ports at a rate of 11 µL/min using a peristaltic perfusion pump, while delivery into the device culture well was achieved via two separate delivery channels with a total combined flow rate of 11 µL/min (stimulus 1 µL/min, containment 10 µL/min). For the characterization experiments, 6% w/v agarose slices were used as a semi-transparent porous matrix to enable visualization of fluorescent media through the slice for adequate characterization. The 10-mm diameter slice was placed over the ports and secured with a washer to prevent slice movement during experimentation.

### Sustained and spatially resolved delivery of stimulus is contained for 1 h

The goal of this work is to provide an easily fabricated and tunable microfluidic platform to deliver localized and sustained stimuli to tissue. The specific objective and proof-of-principle application for our analysis was to confirm that we could contain the stimulus to within a few hundred microns over a period of at least an hour to mimic focal ischemic events in the brain that can occur for several minutes or up to an hour. As mentioned prior, many localized events, like focal ischemia, exist in biology and occur on a variety of timescales and spatial regimes. This application enabled the limits of the device to be examined in terms of spatial resolution of delivery, yet we anticipate the utility of this platform for a variety of equally interesting biological applications ex vivo. To characterize the device’s ability to achieve these goals, we delivered fluorescein to the stimulus delivery ports, at a flow rate of 1 µL/min, while simultaneously delivering non-fluorescent buffer to the surrounding floret ports at 10 µL/min. We have used this approach in prior work to assess the spatial resolution of delivery [26]. To specifically quantitate the containment of the stimulus delivery over time, a series of time-lapse images was taken every 10 s for 1 min, every 1 min for 9 min, and every 10 min for 50 min during the experiment. Example images are shown in Fig. 2A at 1 min, 10 min, and 1 h; qualitatively, there is no difference in the spread of the delivery region over time. Likewise, the images demonstrate the spatial containment in multiple ports simultaneously demonstrating the power of the platform to deliver multiplexed focal and spatially contained stimuli to tissue.

To further quantitate the spatial resolution over time of the delivery on-chip, the spread of the fluorescent signal was calculated as a function of time. Due to the stimulus ports being 100 µm in diameter, any deviation from this port size is considered spread. Spread was calculated by plotting the fluorescent intensity vs. the distance of a line drawn through the center of the fluorescein delivery point. From this line scan, the maximum fluorescence intensity is determined and then divided by half, and the spread was calculated at half-max, similar to our prior work [5] (Figure S1). There was no significant difference between the calculated spread at 1 min and 60 min across each of the four stimulus ports (Fig. 2B, n = 5, One-Way ANOVA with Bonferroni post-tests, *p *= 0.4867), demonstrating that the stimulus delivery can be maintained for at least 1 h in each of the ports. This is particularly advantageous for mimicking longer-term focal events in tissue. For this work, it is specifically important to enable on-site local neurochemical measurements over longer periods of time during local ischemia induction. Previously, our lab reported an average spread of fluorescent signal of 554.2 ± 46 µm at 1 min; here, on average, we only have spread of the fluorescent signal (averaged across all four ports) by 264.1 ± 27 µm, which is an improvement of 53% at the minute mark (*n* = 5). Not only is this a significant improvement in spatial resolution, but with this newly designed chip, we observe that the spatial resolution of delivery is stable over an hour-long period: significantly longer than our prior work. It should be noted that the streaking/shadowing observed in Fig. 2A over time is due to the location of the waste ports, which are positioned at the periphery of the chip (as shown in Fig. 1). These artifacts result from fluorescent waste being pulled toward the outer edges and do not reflect stimulus leakage. As such, they are not included in spatial containment calculations, which focus solely on the central stimulus delivery region. This distinction is critical for accurate interpretation of the containment performance of our microfluidic device.

Containment of spatially resolved delivery is maintained over time. On average, the spread of the stimulus is 304 ± 45 at 60 min. The stable spatial containment can be observed in Fig. 2C; a plot of the average containment size over 60 min at all four ports is demonstrated. This data indicates no significant fluctuations in the containment over the allotted time (One-Way ANOVA with Bonferroni post-tests, *p* = 0.3709, *n* = 5), which will make this device ideal for not only local voltammetric studies but long-term assessment of tissues during focal assault, but for many applications beyond the scope of this paper [39–41]. As a proof-of-principle to demonstrate the power of the device, we also show dual delivery of two different fluorescent dyes (fluorescein and rhodamine) to the stimulus ports with simultaneous delivery of non-fluorescent aCSF to the florets (1:10 flow ratio, Fig. 2D). This further shows the power, locality, and specificity capability of our novel microengineered platform for local and sustained dual delivery. Overall, these results provide evidence that two different stimuli can be administered to tissue ex vivo. We anticipate that this could be particularly useful for delivering two different pharmacological agents to tissue to study fundamental mechanisms of cell signaling experiencing focal injury.

### Viability of brain tissue on-chip

**Fig. 3:**
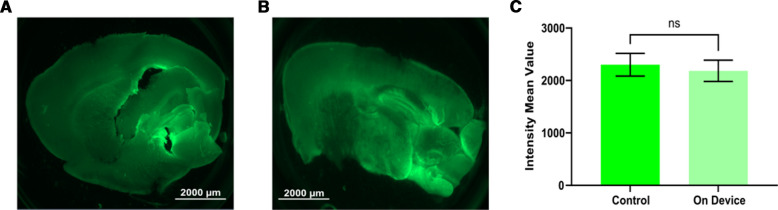
Brain slices remain viable on-chip compared to standard perfusion methods. **A** Tissue from a standard perfusion chamber with global delivery of aCSF demonstrating our device is similar in maintaining tissue viability for voltammetric analysis. **B** Tissue from our 3D device after delivery of aCSF through all ports. **C** Intensity mean value compared across **A** and **B**; there are no quantifiable changes between tissues (unpaired *t*-test, *p* = 0.7149, *n* = 5)

Tissue remains viable on board the device over time, demonstrating its applicability for acute ex vivo studies. To examine the viability of the tissue on the device, sagittal brain slices (*n* = 5) were placed on the platform for 1 h, the typical length of a voltammetry experiment [42], and given the necessary oxygenated aCSF for hemostasis to be maintained (standard brain slice culture) [39]. After an hour, slices were stained using Calcein-AM. For a positive control (*n* = 5), tissue was placed in a standard perfusion chamber used for neurochemical analysis, given the necessary buffer bath over the hour period, then also stained with Calcein-AM for a direct comparison of on- versus off-chip. Calcein-AM can integrate intercellularly and interact with esterases within the tissue [43–45]. Since it is known that dead cells do not contain esterase complexes, only living cells will fluoresce green [38]. Figure 3B indicates that tissue is viable on the platform for at least an hour, and these results are comparable to a standard perfusion chamber (Fig. 3A). This conclusion was made due to the relative fluorescent intensity in both the on- and off-chip cohorts not being statistically different (Fig. 3C). Further investigations could analyze the longevity of the culture on-chip for different studies; however, this experiment shows that for this work, the necessary cellular survival rate is ensured.

**Fig. 4:**
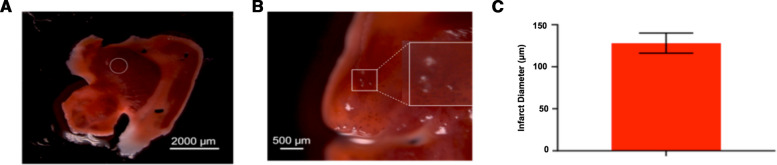
TTC staining reveals that local damage from a focal ischemic assault can be delivered to brain tissue on-chip. **A** An example image of a sagittal slice after local delivery of ischemic buffer (slice 1). The circle shows where local delivery was administered. **B** Additional example demonstrating a magnified area of a localized delivery region (slice 2). The delivery spot is further magnified within the inset. **C** The average infarct diameter calculated at the localized delivery points is 128 ± 15 µm (*n* = 5 slices), demonstrating near complete containment of the focal stimulus in the tissue

Local tissue injury was validated on-chip using TTC staining. TTC staining was chosen due to its ability to analyze regions of local injury. TTC enables visualization of infarcted regions across tissue sections [46–48]. If the tissue is metabolically active, it will be stained red; however, if the tissue is metabolically inactive and damaged, it will be stained white/tissue color [46–48]. In this experiment, aCSF was delivered through the buffer channel/florets to maintain optimal tissue health and to contain the stimulus delivery. Ischemic media (aCSF lacking oxygen and glucose [39, 49, 50]) was delivered through the stimulus ports with simultaneous containment for 30 min in slices of hippocampus on-chip (*n* = 5). In a separate cohort (*n* = 5) as a control, normal aCSF was delivered through all ports. Tissue cohorts were then taken off the devices, and the TTC staining protocol was followed (see methods). Figure 4A and B shows two example slices after onboard the device. A small, localized infarcted region of damage, corresponding to the delivery regions, is shown (Fig. 4A, white circle, and Fig. 4B, white box). The region of interest in Fig. 4B is further magnified to demonstrate the small local white regions, corresponding to decreased metabolic activity at the stimulation zones. On average, the infarcted regions were quantitated to be 128 ± 15 µm (*n* = 5) in diameter. The stimulation port is 100 µm in diameter; therefore, only a ~ 28-µm deviation from this port of damage was observed. We suspect that the improved containment of delivery observed in tissue, compared to delivery to agarose in the prior experiments, is a direct result of the different permeabilities and porosities of these materials (see control experiments in Figure S2A: these experiments show no infarct tissue damage on the device). Thus, this demonstrates the feasibility of our device to deliver a localized, sustained chemical injury to living brain tissue.

### Neurochemical signaling at the point of local injury

The microengineered 3D platform is designed as open-welled to facilitate external probing for precise local chemical monitoring at the site of focal assault. Specifically in this work, we have incorporated carbon-fiber microelectrodes for local electrochemical monitoring of neurochemical signaling during focal ischemia. Here, we implanted a carbon-fiber microelectrode directly within the tissue over the stimulus region, and the reference electrode was placed in the bath nearby. Fast-scan cyclic voltammetry (FSCV) was used to measure real-time fluctuations of dopamine signaling at the site of focal injury, similar to our prior work [26, 49, 50]. Dopamine signaling at the site of focal assault in the brain is not well understood due to current limitations in technology to enable these measurements. We were the first to report focal dopamine fluctuations during ischemia in our prior work, and here, we provide further evidence that there exists a rapid and sustained change in dopaminergic signaling during focal ischemia. Measuring chemical signaling on-chip also further validates that tissues remain functional on the device. Dopamine proves to be a great biomarker for ischemia due to the fact that it is the standard for FSCV detection [53, 54], and it has also been shown to have large changes in endogenous release during ischemic conditions, yet the nuances in how it signals locally at the injury site are unknown [31, 55–57].

**Fig. 5:**
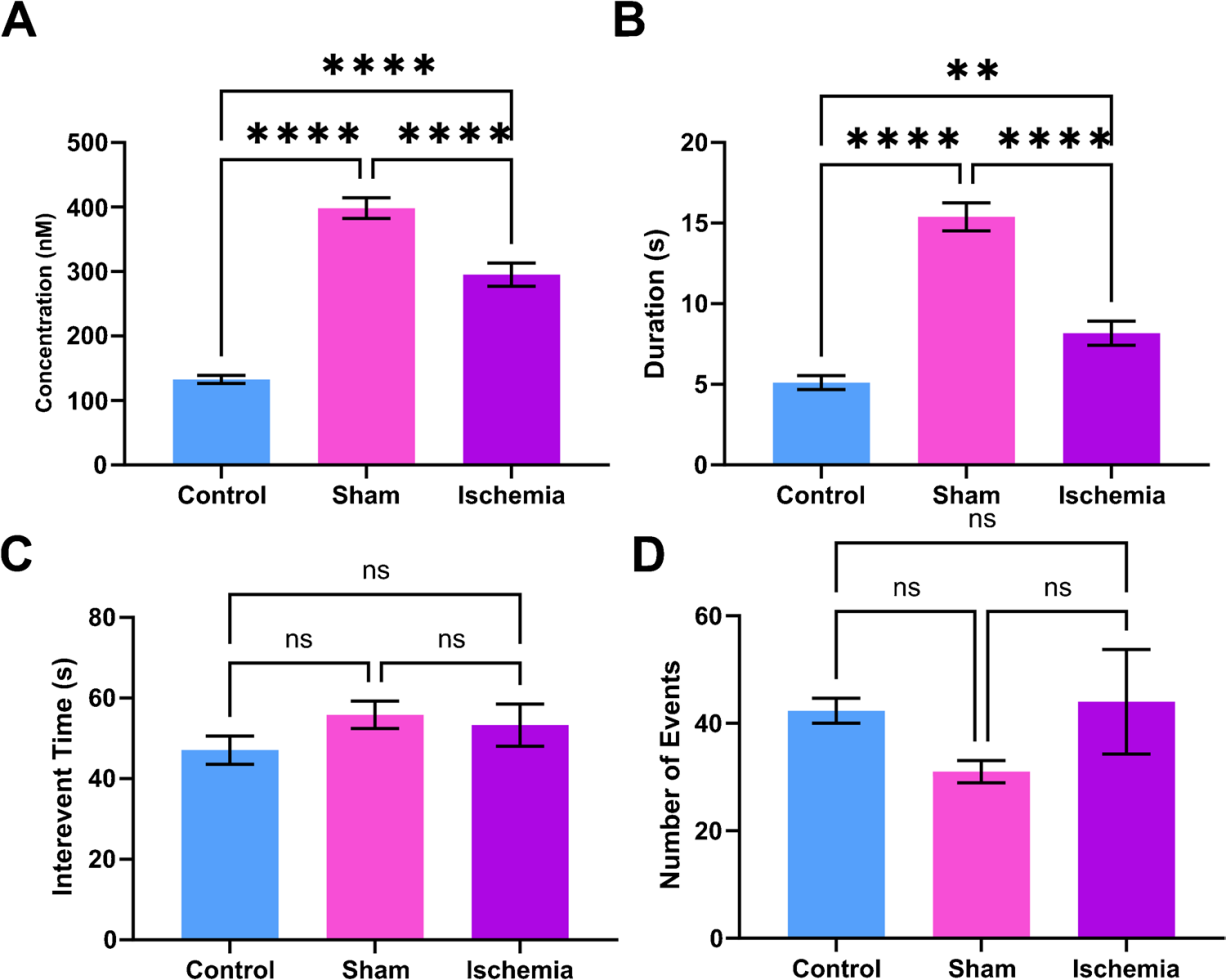
Focal ischemic conditions on-chip induce increased dopamine signaling. **A** Transient concentrations increase following ischemic conditions. **B** Signal duration also increases significantly, more than doubling on average. **C** Frequency of release remains unchanged in focal ischemia conditions. **D** The number of events also remains stable during focal ischemia (*n* = 3). One-way ANOVA with Bonferroni post-tests, *p* = 0.4867

Dynamic dopamine signaling was monitored in slices for 50 min after electrode implantation, and data were recorded for 45 min, allowing a 5-min acclimation. Each slice was divided into three 15-min sections, with the first 15 min as the control and the last 15 min as the treatment. The ischemia buffer was constantly perfused for the last 30 min of measurement to the center point of the “floret” (the stimulus port), enabling a 15-min equilibration period before comparison of control and treatment groups. The incubation period of ischemic buffer was also quantified and is labeled as “sham” throughout the data sets below. Transient event concentration, duration, frequency (or interevent time), and number of events were quantified. Concentration was determined via a previously set calibration factor, duration was determined by measuring the time between the current half-maximum at the beginning and end of the transient, and interevent time was the period between the end of one transient and the start of the subsequent event, similar to many prior reports quantitating transient neurochemical signaling with FSCV [11, 22, 26, 38]. Overall, a significant increase in dopamine transient concentration and event duration, giving rise to an overall increase in extracellular dopamine content (Fig. 5, *n* = 3), was observed at the site of focal ischemia on-chip. During ischemic assault, there is an expected increase in extracellular dopamine correlated with cell death in the affected ischemic area [9, 26, 51, 52]. Future work could focus on investigating the local mechanism of dopamine signaling at the site and as a function of ischemic severity on-chip.

## Conclusion

This paper has presented a novel, 3D-printed microfluidic platform to induce focal delivery of stimulus to tissues ex vivo. This demonstrates a marked improvement in prior approaches, demonstrating the novelty and high impact of the work. We report a 53% improvement in spatial resolution maintained over time at the 1-min mark compared to our previously published work. However, most advantageously, there is an increase in the length of time the stimulus is spatially contained (1 h), further broadening the experimental possibilities. We anticipate the utility of this platform for future pharmacology studies to understand local pharmacodynamics with the platform’s dual delivery system during normal or chemically induced stimulation in tissue. Our specific application of measuring local neurochemical events during focal injury provides not only an important proof-of-concept, but also an interesting segue into understanding dopamine’s complex signaling modalities during focal injury. Ultimately, our new device can be used to deliver local chemical injury to living tissue and probe the real-time release of neurochemicals at the site of damage, thus bridging a critical gap in the literature to understand local physiology due to ischemic injury. Though the application that we chose to explore with this 3D-printed approach for spatial containment was focal ischemia, this method can be used further to explore a plethora of other biological problems that need to be explored on a spatially resolved and dynamic time scale.

## Supplementary Information

Below is the link to the electronic supplementary material.

Supplementary Material 1 (DOCX 400 KB)

## Data Availability

Data will be made available upon request.

## References

[1] Dai W-J, Zhu L-Y, Yan Z-Y, Xu Y, Wang Q-L, Lu X-J. CRISPR-Cas9 for in vivo gene therapy: promise and hurdles. Mol Ther Nucleic Acids. 2016;5(8):e349. 10.1038/mtna.2016.58. 28131272 10.1038/mtna.2016.58 PMC5023403

[2] Precise genome-editing in human diseases: mechanisms, strategies and applications | Signal Transduction and Targeted Therapy. https://www.nature.com/articles/s41392-024-01750-2. Accessed 12–14–2024. 10.1038/s41392-024-01750-2 PMC10897424 38409199

[3] Hanahan D, Weinberg RA. Hallmarks of cancer: the next generation. Cell. 2011;144(5):646–74. 10.1016/j.cell.2011.02.013. 21376230 10.1016/j.cell.2011.02.013

[4] Kuchmiy AA, Efimov GA, Nedospasov SA. Methods for in vivo molecular imaging. Biochemistry (Moscow). 2012;77(12):1339–53. 10.1134/S0006297912120012. 23244729 10.1134/S0006297912120012

[5] A microfluidic chip for sustained oxygen gradient formation in the intestine ex vivo - Lab on a Chip (RSC Publishing). https://pubs.rsc.org/en/content/articlelanding/2024/lc/d3lc00793f. Accessed 06–03–2024. 10.1039/d3lc00793f PMC10998727 38372633

[6] Sommer CJ. Ischemic stroke: experimental models and reality. Acta Neuropathol. 2017;133(2):245–61. 10.1007/s00401-017-1667-0. 28064357 10.1007/s00401-017-1667-0 PMC5250659

[7] Cho S, Wood A, Bowlby MR. Brain slices as models for neurodegenerative disease and screening platforms to identify novel therapeutics. Curr Neuropharmacol. 2007;5(1):19–33. 10.2174/157015907780077105. 18615151 10.2174/157015907780077105 PMC2435340

[8] Sarntinoranont M, Lee SJ, Hong Y, King MA, Subhash G, Kwon J, et al. High-strain-rate brain injury model using submerged acute rat brain tissue slices. J Neurotrauma. 2012;29(2):418–29. 10.1089/neu.2011.1772. 21970544 10.1089/neu.2011.1772

[9] Opitz A, Falchier A, Linn GS, Milham MP, Schroeder CE. Limitations of ex vivo measurements for in vivo neuroscience. Proceedings of the National Academy of Sciences. 2017;114(20):5243–6. 10.1073/pnas.1617024114. 10.1073/pnas.1617024114 PMC5441777 28461475

[10] Andersson H, den Berg A. Microfabrication and microfluidics for tissue engineering: state of the art and future opportunities. Lab Chip. 2004;4(2):98–103. 10.1039/B314469K. 15052347 10.1039/b314469k

[11] Duncombe TA, Tentori AM, Herr AE. Microfluidics: reframing biological enquiry. Nat Rev Mol Cell Biol. 2015;16(9):554–67. 10.1038/nrm4041. 26296163 10.1038/nrm4041 PMC6240156

[12] Ross AE, Belanger MC, Woodroof JF, Pompano RR. Spatially resolved microfluidic stimulation of lymphoid tissue ex vivo. Analyst. 2017;142(4):649–59. 10.1039/C6AN02042A. 27900374 10.1039/c6an02042a PMC7863610

[13] Liu F, McCullough LD. Middle cerebral artery occlusion model in rodents: methods and potential pitfalls. Biomed Res Int. 2011;2011(1):464701. 10.1155/2011/464701. 10.1155/2011/464701 PMC3035178 21331357

[14] Cryan MT, Li Y, Ross AE. Sustained delivery of focal ischemia coupled to real-time neurochemical sensing in brain slices. Lab Chip. 2022;22(11):2173–84. 10.1039/d1lc00908g. 35531656 10.1039/d1lc00908g PMC9156565

[15] Whitesides GM. The origins and the future of microfluidics. Nature. 2006;442(7101):368–73. 10.1038/nature05058. 16871203 10.1038/nature05058

[16] Catterton MA, Dunn AF, Pompano RR. User-defined local stimulation of live tissue through a movable microfluidic port. Lab Chip. 2018;18(14):2003–12. 10.1039/C8LC00204E. 29904762 10.1039/c8lc00204e PMC6039252

[17] Mauleon G, Fall CP, Eddington DT. Precise spatial and temporal control of oxygen within in vitro brain slices via microfluidic gas channels. PLoS One. 2012;7(8):e43309. 10.1371/journal.pone.0043309. 22905255 10.1371/journal.pone.0043309 PMC3419219

[18] Delong LM, Witt CE, Pennell M, Ross AE. A microfluidic chip for sustained oxygen gradient formation in the intestine ex vivo. Lab Chip. 2024;24(7):1918–29. 10.1039/D3LC00793F. 38372633 10.1039/d3lc00793f PMC10998727

[19] Types of Strokes | SpringerLink. 10.1007/978-1-62703-380-0_2. Accessed 06–09–2024.

[20] Smith WS. Ischemic stroke. Curr Opin Crit Care. 1998;4(2):89–93. 10.1097/00075198-199804000-00004.

[21] Favate AS, Younger DS. Epidemiology of ischemic stroke. Neurol Clin. 2016;34(4):967–80. 10.1016/j.ncl.2016.06.013. 27720004 10.1016/j.ncl.2016.06.013

[22] Plummer P, Behrman AL, Duncan PW, Spigel P, Saracino D, Martin J, et al. Effects of stroke severity and training duration on locomotor recovery after stroke: a pilot study. Neurorehabil Neural Repair. 2007;21(2):137–51. 10.1177/1545968306295559. 17312089 10.1177/1545968306295559

[23] Brocklehurst JC, Morris P, Andrews K, Richards B, Laycock P. Social effects of stroke. Social Science & Medicine Part A: Medical Psychology & Medical Sociology. 1981;15(1):35–9. 10.1016/0271-7123(81)90043-2. 10.1016/0271-7123(81)90043-2 6973195

[24] Dorrance, A. M.; Fink, G. Effects of stroke on the autonomic nervous system. In *Comprehensive Physiology*; Terjung, R., Ed.; Wiley, 2015; pp 1241–1263. 10.1002/cphy.c140016. 10.1002/cphy.c140016 26140717

[25] Neurochemical changes underpinning the development of adjunct therapies in recovery after stroke: a role for GABA? - Ainslie Johnstone, Jacob M Levenstein, Emily L Hinson, Charlotte J Stagg, 2018. 10.1177/0271678X17727670. Accessed 06–09–2024. PMC6125966 28929902

[26] Alves, P.; Nozais, V.; Hansen, J.; Corbetta, M.; Nachev, P.; Martins, I.; Schotten, M. T. de. Neurotransmitters’ white matter mapping unveils the neurochemical fingerprints of stroke. February 21, 2024. 10.21203/rs.3.rs-3937453/v1. 10.1038/s41467-025-57680-2 PMC11910582 40089467

[27] Yan, Y. C. L. The dopaminergic mechanisms underlying ischemic stroke recovery and motor learning. Ph.D., The Chinese University of Hong Kong (Hong Kong), Hong Kong. https://www.proquest.com/docview/2070596511/abstract/36512A6E449A486DPQ/1. Accessed 06–09–2024.

[28] Witt CE, Mena S, Holmes J, Hersey M, Buchanan AM, Parke B, et al. Serotonin is a common thread linking different classes of antidepressants. Cell Chem Biol. 2023;30(12):1557-1570.e6. 10.1016/j.chembiol.2023.10.009. 37992715 10.1016/j.chembiol.2023.10.009

[29] Ross AE, Venton BJ. Adenosine transiently modulates stimulated dopamine release in the caudate-putamen via A1 receptors. J Neurochem. 2015;132(1):51–60. 10.1111/jnc.12946. 25219576 10.1111/jnc.12946 PMC4270927

[30] Rudolph M, Schmeer CW, Günther M, Woitke F, Kathner-Schaffert C, Karapetow L, et al. Microglia-mediated phagocytosis of apoptotic nuclei is impaired in the adult murine hippocampus after stroke. Glia. 2021;69(8):2006–22. 10.1002/glia.24009. 33942391 10.1002/glia.24009

[31] Toner CC, Connelly K, Whelpton R, Bains S, Michael-Titus AT, McLaughlin DP, et al. Effects of sevoflurane on dopamine, glutamate and aspartate release in an in vitro model of cerebral ischaemia. Br J Anaesth. 2001;86(4):550–4. 10.1093/bja/86.4.550. 11573631 10.1093/bja/86.4.550

[32] Duration of glutamate release after acute ischemic stroke | Stroke. 10.1161/01.STR.28.4.708. Accessed 06–09–2024. 9099183

[33] Dopaminergic innervation and modulation of hippocampal networks | Cell and Tissue Research. 10.1007/s00441-018-2800-7. Accessed 06–09–2024. 29470647

[34] Dohmen C, Sakowitz OW, Fabricius M, Bosche B, Reithmeier T, Ernestus R-I, et al. Spreading depolarizations occur in human ischemic stroke with high incidence. Ann Neurol. 2008;63(6):720–8. 10.1002/ana.21390. 18496842 10.1002/ana.21390

[35] Mechanisms of neuronal cell death in ischemic stroke and their therapeutic implications - Tuo - 2022 - Medicinal Research Reviews - Wiley Online Library. 10.1002/med.21817. Accessed 06–09 –2024. 33957000

[36] Bath BD, Michael DJ, Trafton BJ, Joseph JD, Runnels PL, Wightman RM. Subsecond adsorption and desorption of dopamine at carbon-fiber microelectrodes. Anal Chem. 2000;72(24):5994–6002. 10.1021/ac000849y. 11140768 10.1021/ac000849y

[37] Ross AE, Pompano RR. Diffusion of cytokines in live lymph node tissue using microfluidic integrated optical imaging. Anal Chim Acta. 2018;1000:205–13. 10.1016/j.aca.2017.11.048. 29289312 10.1016/j.aca.2017.11.048

[38] Sadeh N, Oni-Biton E, Segal M. Acute live/dead assay for the analysis of toxic effects of drugs on cultured neurons. BIO-Protoc. 2016;6(15). 10.21769/BioProtoc.1889.

[39] Weese-Myers ME, Cryan MT, Witt CE, Caldwell KCN, Modi B, Ross AE. Dynamic and rapid detection of guanosine during ischemia. ACS Chem Neurosci. 2023;14(9):1646–58. 10.1021/acschemneuro.3c00048. 37040534 10.1021/acschemneuro.3c00048 PMC10265669

[40] Hensley AL, Colley AR, Ross AE. Real-time detection of melatonin using fast-scan cyclic voltammetry. Anal Chem. 2018;90(14):8642–50. 10.1021/acs.analchem.8b01976. 29932641 10.1021/acs.analchem.8b01976

[41] Lim GN, Regan SL, Ross AE. Subsecond spontaneous catecholamine release in mesenteric lymph node ex vivo. J Neurochem. 2020;155(4):417–29. 10.1111/jnc.15115. 32602936 10.1111/jnc.15115

[42] Measuring neuron-regulated immune cell physiology via the alpha-2 adrenergic receptor in an ex vivo murine spleen model | Cellular and Molecular Life Sciences. 10.1007/s00018-023-05012-2. Accessed 06–09–2024. PMC11071927 37945921

[43] Neri S, Mariani E, Meneghetti A, Cattini L, Facchini A. Calcein-acetyoxymethyl cytotoxicity assay: standardization of a method allowing additional analyses on recovered effector cells and supernatants. Clin Diagn Lab Immunol. 2001;8(6):1131–5. 10.1128/cdli.8.6.1131-1135.2001. 11687452 10.1128/CDLI.8.6.1131-1135.2001 PMC96238

[44] Tenopoulou M, Kurz T, Doulias P-T, Galaris D, Brunk UT. Does the calcein-AM method assay the total cellular ‘labile iron pool’ or only a fraction of it? Biochem J. 2007;403(2):261–6. 10.1042/BJ20061840. 17233627 10.1042/BJ20061840 PMC1874234

[45] Uggeri J, Gatti R, Belletti S, Scandroglio R, Corradini R, Rotoli BM, et al. Calcein-AM is a detector of intracellular oxidative activity. Histochem Cell Biol. 2000;122(5):499–505. 10.1007/s00418-004-0712-y. 10.1007/s00418-004-0712-y 15503120

[46] Triphenyltetrazolium chloride (TTC) as a marker for ischaemic changes in rat brain following permanent middle cerebral artery occlusion - HATFIELD - 1991 - Neuropathology and Applied Neurobiology - Wiley Online Library. 10.1111/j.1365-2990.1991.tb00694.x. Accessed 06–09–2024. 2057051

[47] Ruiz-Crespo S, Trejo-Gabriel-Galán JM, Coma-del-Corral MJ. Localizing coordinates of cerebral ischemic tissue without the need of staining in a rat model of focal cerebral infarct. Metab Brain Dis. 2013;28(1):21–4. 10.1007/s11011-012-9359-x. 23160835 10.1007/s11011-012-9359-x

[48] Li Z, Bishop N, Chan S-L, Cipolla MJ. Effect of TTC treatment on immunohistochemical quantification of collagen IV in rat brains after stroke. Transl Stroke Res. 2018;9(5):499–505. 10.1007/s12975-017-0604-9. 29313240 10.1007/s12975-017-0604-9 PMC6035895

[49] Thomaz DT, Andreguetti RR, Binder LB, Scheffer DdaL, Corrêa AW, Silva FRMB, et al. Guanosine neuroprotective action in hippocampal slices subjected to oxygen and glucose deprivation restores ATP levels, lactate release and glutamate uptake impairment: involvement of nitric oxide. Neurochem Res. 2020;45(9):2217–29. 10.1007/s11064-020-03083-2. 32666283 10.1007/s11064-020-03083-2

[50] Dal-Cim T, Ludka FK, Martins WC, Reginato C, Parada E, Egea J, et al. Guanosine controls inflammatory pathways to afford neuroprotection of hippocampal slices under oxygen and glucose deprivation conditions. J Neurochem. 2013;126(4):437–50. 10.1111/jnc.12324. 23713463 10.1111/jnc.12324

[51] Jill Venton B, Cao Q. Fundamentals of fast-scan cyclic voltammetry for dopamine detection. Analyst. 2020;145(4):1158–68. 10.1039/C9AN01586H. 31922176 10.1039/c9an01586h PMC7028514

[52] Ou Y, Marie Buchanan A, E. Witt C, Hashemi P. Frontiers in electrochemical sensors for neurotransmitter detection: towards measuring neurotransmitters as chemical diagnostics for brain disorders. Anal Methods. 2019;11(21):2738–55. 10.1039/C9AY00055K. 32724337 10.1039/c9ay00055k PMC7386554

[53] Brannan T, Weinberger J, Knott P, Taff I, Kaufmann H, Togasaki D, et al. Direct evidence of acute, massive striatal dopamine release in gerbils with unilateral strokes. Stroke. 1987;18(1):108–10. 10.1161/01.str.18.1.108. 3810742 10.1161/01.str.18.1.108

[54] Oliva I, Fernández M, Martín ED. Dopamine release regulation by astrocytes during cerebral ischemia. Neurobiol Dis. 2013;58:231–41. 10.1016/j.nbd.2013.06.007. 23800715 10.1016/j.nbd.2013.06.007

[55] Saulle E, Centonze D, Martín AB, Moratalla R, Bernardi G, Calabresi P. Endogenous dopamine amplifies ischemic long-term potentiation via D1 receptors. Stroke. 2002;33(12):2978–84. 10.1161/01.str.0000038093.42512.0f. 12468800 10.1161/01.str.0000038093.42512.0f

[56] Furukawa N, Arai N, Goshima Y, Miyamae T, Ohshima E, Suzuki F, et al. Endogenously released DOPA is a causal factor for glutamate release and resultant delayed neuronal cell death by transient ischemia in rat striata. J Neurochem. 2001;76(3):815–24. 10.1046/j.1471-4159.2001.00068.x. 11158253 10.1046/j.1471-4159.2001.00068.x

[57] Buisson A, Callebert J, Mathieu E, Plotkine M, Boulu RG. Striatal protection induced by lesioning the substantia nigra of rats subjected to focal ischemia. J Neurochem. 1992;59(3):1153–7. 10.1111/j.1471-4159.1992.tb08358.x. 1353789 10.1111/j.1471-4159.1992.tb08358.x

