# Supplementary material for: Engineering localized injury: a 3D microfluidic platform approach for ex vivo tissue interrogation
Source: Anal Bioanal Chem. 2025 Dec 6;418(15):4735–47. doi: 10.1007/s00216-025-06245-9 (PMC13388642; doi:10.1007/s00216-025-06245-9)
Supplement: Supplementary file 1 — Supplementary Material 1 (DOCX 400 KB) [file 216_2025_6245_MOESM1_ESM.docx]

Supplemental File:

Engineering Localized Injury: A 3D Microfluidic Platform Approach for *Ex Vivo* Tissue Interrogation

Colby E. Witt^1^, Lauren M. Delong^1^, Maria K. Kristinsdottir^1^, Alexandra K. Brooke ^1^, and Ashley E. Ross^1*^

^1^University of Cincinnati

Department of Chemistry

312 College Dr.

404 Crosley Tower

Cincinnati, OH 45221-0172, USA

Office Phone#: 513-556-9314

Email: [Ashley.ross@uc.edu](mailto:Ashley.ross@uc.edu)

*Corresponding author

*Colby E. Witt 0000-0002-7332-7632*

*Lauren M. Delong 0000-0002-1266-3536*

*Maria K. Kristindottir*

*Alexandra K. Brooke 0000-0003-2440-4758*

*Ashley E. Ross 0000-0003-2456-3636*

**Key words:** Ischemia, neurochemicals, tissue stimulation, microfluidics, microfabrication


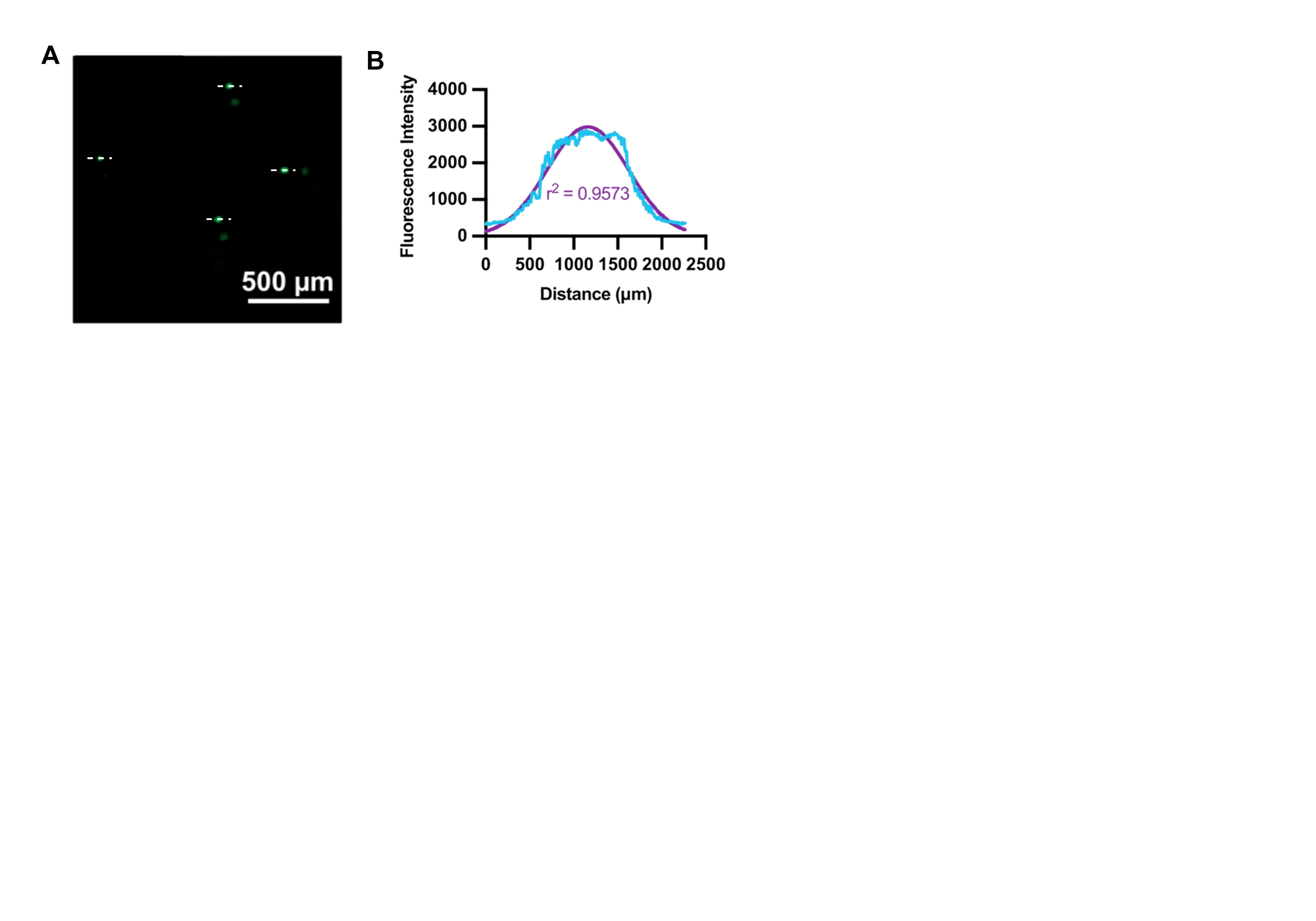


**Figure S1:** Gradient size is quantitated on-chip using fluorescence microscopy. (A) Image of fluorescein delivery to an agarose slice on-chip. Fluorescein media was perfused through the middle “deoxygenated delivery ports” while normal media was delivered through the “oxygenated delivery ports”. (B) Example linescan. Data was taken from the dashed line in A. From this data spread and the distance at half maximum intensity was calculated.


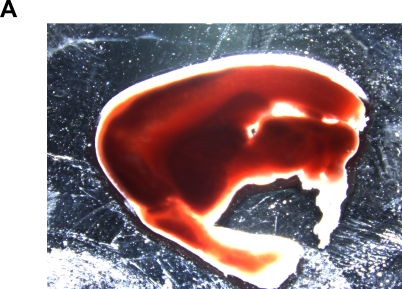


**Figure S2:** TTC staining on rat brain tissue with no ischemic damage delivered to tissue from the analyte ports on the device. No damage can be seen when compared to figure 3 in the main text.
